# Inhibiting Heat Shock Protein 90 Protects Nucleus Pulposus-Derived Stem/Progenitor Cells From Compression-Induced Necroptosis and Apoptosis

**DOI:** 10.3389/fcell.2020.00685

**Published:** 2020-08-07

**Authors:** Binwu Hu, Shuo Zhang, Weijian Liu, Peng Wang, Songfeng Chen, Xiao Lv, Deyao Shi, Kaige Ma, Baichuan Wang, Yongchao Wu, Zengwu Shao

**Affiliations:** ^1^Department of Orthopaedics, Union Hospital, Tongji Medical College, Huazhong University of Science and Technology, Wuhan, China; ^2^Department of Orthopaedic Surgery, The First Affiliated Hospital of Zhengzhou University, Zhengzhou, China

**Keywords:** nucleus pulposus-derived stem/progenitor cells, heat shock protein 90, necroptosis, apoptosis, intervertebral disc degeneration, compression

## Abstract

Nucleus pulposus-derived stem/progenitor cells (NPSCs) provide novel prospects for the regeneration of degenerated intervertebral disc (IVD). Nevertheless, with aging and degeneration of IVD, the frequency of NPSCs markedly decreases. Excessive cell death could be the main reason for declined frequency of NPSCs, however, the exact mechanisms remain elusive. Thus, the present study was undertaken to explore the mechanisms of compression-induced NPSCs death, and the effects of heat shock protein 90 (HSP90) on NPSCs survival. Here, we found that compression could trigger receptor-interacting protein kinase 1 (RIPK1)/receptor-interacting protein kinase 3 (RIPK3)/mixed lineage kinase domain-like protein (MLKL)-mediated necroptosis of NPSCs. Furthermore, we found that elevated expression of HSP90 was involved in compression-induced NPSCs death, and inhibiting HSP90 could dramatically attenuate compression-induced necroptosis of NPSCs via regulating the expression and activity of RIPK1/RIPK3/MLKL, and alleviating the mitochondrial dysfunction (mitochondrial membrane potential loss and ATP depletion) and oxidative stress [production of mitochondrial reactive oxygen species (ROS), cellular total ROS and malondialdehyde, and downregulation of superoxide dismutase 2]. Besides necroptosis, compression-induced apoptosis of NPSCs was also attenuated by HSP90 inhibition. In addition, we found that enhanced expression of HSP70 contributed to the cytoprotective effects of inhibiting HSP90. More encouragingly, our results demonstrated that inhibiting HSP90 could also mitigate the exhaustion of NPSCs *in vivo*. In conclusion, RIPK1/RIPK3/MLKL-mediated necroptosis participates in compression-induced NPSCs death. Furthermore, targeting HSP90 to simultaneously inhibit necroptosis and apoptosis of NPSCs might be an efficient strategy for preventing the death of NPSCs, thus rescuing the endogenous repair capacity of NP tissue.

## Introduction

Intervertebral disc (IVD) degeneration (IVDD) has been widely considered as the paramount etiology for low back pain, leading to heavy socio-economic burden due to lost productivity and increasing health care costs (Maetzel and Li, 2002; Freemont, 2009). Accumulating evidence has demonstrated that dysregulation of the function and quantity of nucleus pulposus (NP) cells accounts for the initiation of IVDD (Buckwalter, 1995; Li et al., 2018b). Therefore, many treatments aiming at retarding or reversing disc degeneration, including cell transplantation, growth factors supplementation and gene therapy, mainly focus on reviving the function and quantity of NP cells (Smith et al., 2011). However, these established methods have limitations more or less (Zhao et al., 2007). Recently, many studies have proven the existence of NP-derived stem/progenitor cells (NPSCs) (Risbud et al., 2007; Hu and He, 2018). It is reported that NPSCs could secrete extracellular matrix and differentiate toward NP cells (Tao et al., 2015). Furthermore, the co-culture of NP cells and NPSCs could enhance cell proliferation and the expression of chondrogenic-associated genes under hypertonicity condition (Tao et al., 2013). Thus, NPSCs might provide novel prospects for the regeneration of degenerated IVD. However, with aging and degeneration of IVD, the frequency of NPSCs markedly decreases, indicating the exhaustion of NPSCs (Sakai et al., 2012). Excessive cell death could be the main reason for the exhaustion of NPSCs. Previous studies have demonstrated that many adverse microenvironment factors, such as high osmolarity, low pH, oxidative stress and mechanical loading, could induce NPSCs death (Hu and He, 2018; Nan et al., 2019). Nevertheless, the precise mechanisms have not yet been fully elucidated.

Mechanical loading has long been recognized as the leading cause of IVDD (Neidlinger-Wilke et al., 2014). Our previous studies demonstrate that programmed cell death, such as apoptosis and necroptosis, participates in compression-induced NP cells death (Ding et al., 2012; Chen et al., 2017). Meanwhile, we found that compression could also trigger apoptosis of NPSCs (Li et al., 2018a). However, it has not been fully elucidated whether other types of programmed cell death are involved in compression-induced NPSCs death. Necroptosis is a newly defined form of regulated necrosis, which is mainly mediated by receptor-interacting protein kinase 1 (RIPK1)/receptor-interacting protein kinase 3 (RIPK3)/mixed lineage kinase domain-like protein (MLKL) signaling pathway (Hu et al., 2018). It has been reported that necroptosis contributes to various pathophysiological conditions, including systemic inflammation, ischemic reperfusion injury and neurodegeneration (Zhou and Yuan, 2014; Galluzzi et al., 2017). Therefore, unraveling the role of necroptosis in compression-induced NPSCs death, and its regulatory mechanisms might provide novel targets for preventing NPSCs death.

Heat shock protein 90 (HSP90) is a widely-expressed molecular chaperone, which is involved in numerous physiological processes such as signal transduction, intracellular transport, and protein degradation (Li and Buchner, 2013). Generally, HSP90 is thought to maintain cell viability and prevent apoptosis. It is reported that HSP90 could directly promote cell survival through the activation of nuclear factor-kappa B, indirectly promote cell survival via HSP90-Akt complexes, and modulate the intrinsic pathway of apoptosis (Arya et al., 2007). On the contrary, elevated expression of HSP90 could also exert pro-death roles under some circumstances (Hooven et al., 2004; Wang et al., 2011; Ma et al., 2015). Correspondingly, inhibiting HSP90 by specific inhibitors or siRNAs is able to downregulate some cell death-related pathways, such as the IKK/IκB/p65 pathway, as well as activate multiple cell survival pathways, including induction of heat shock protein 70 (HSP70) and the phosphorylation of Akt, extracellular signal regulated kinase 1/2 and glycogen synthase kinase 3β, ultimately attenuating cell death in many pathological process (Wang et al., 2011; Alani et al., 2014; Li J. et al., 2015; Liu Q. et al., 2016). Traditionally, decreased cell death caused by HSP90 inhibition is mainly attributed to reduced cell apoptosis, recent studies also reveal that RIPK1, RIPK3, and MLKL, the critical components of necroptosis signaling pathway, are client proteins of HSP90 (Lewis et al., 2000; Li D. et al., 2015; Zhao et al., 2016). Inhibiting HSP90 could block necroptosis by modulating the stability and function of RIPK1, RIPK3, and MLKL (Yang and He, 2016). Thus, HSP90 could simultaneously regulate apoptosis and necroptosis, which might be a wonderful target for preventing cell death. However, the roles of HSP90 in compression-induced NPSCs death have not been determined yet.

Mitochondrial dysfunction and reactive oxygen species (ROS) are critical in the execution of necroptosis (Marshall and Baines, 2014). Furthermore, they could also trigger cell apoptosis through mitochondrial apoptosis pathway (Feng et al., 2017). Our group has demonstrated that mitochondrial dysfunction and oxidative stress contribute greatly to compression-induced NP cells apoptosis and necroptosis (Ding et al., 2012; Chen et al., 2018). Furthermore, we found that compression could also trigger mitochondrial dysfunction and oxidative stress of NPSCs, leading to cell apoptosis (Li et al., 2018a). Therefore, suppressing mitochondrial dysfunction and oxidative stress might be efficient methods for preventing compression-induced NPSCs apoptosis and necroptosis.

In present study, we first investigated the involvement of necroptosis in compression-induced death of NPSCs. Subsequently, we determined the roles of HSP90 in compression-induced NPSCs death and explored relevant mechanisms. In addition, we also evaluated whether inhibiting HSP90 alleviated the exhaustion of NPSCs *in vivo.* Our study provides novel insights into the mechanisms of compression-induced NPSCs death as well as experimental evidence of a possible target for protecting NPSCs.

## Materials and Methods

### Samples Collection

Experimental protocols for this study were approved by medical ethics committee of Tongji Medical College, Huazhong University of Science and Technology. Written informed consent was obtained from all patients. Human NP tissues were obtained from patients undergoing routine surgery for lumbar and separated by an experienced surgeon in the Department of Orthopaedics, Union Hospital (Wuhan, China). Magnetic resonance images were used to evaluate the degree of IVDD according to Pfirrmann magnetic resonance images-grade system, and the samples of Pfirrmann grade I and II were regarded as non-degenerated (Pfirrmann et al., 2001). All samples were sectioned for the following experiments: (1) for histological analysis, tissues were fixed in 4% formaldehyde and embedded in paraffin, and (2) for cell culture, tissues were immediately immersed in phosphate-buffered saline (PBS).

### Isolation and Culture of Human NPSCs

Human NPSCs were isolated and cultured in accordance with previously established protocols (Liu et al., 2017; Li et al., 2018a; Liang et al., 2018). Briefly, after being washed with PBS for three times, NP tissues were carefully examined using a dissecting microscope to remove any adherent tissues such as the annulus fibrosus, cartilage endplate and ligaments. Subsequently, the NP tissues were minced into small fragments and digested in 0.2% (m/v) type II collagenase (Sigma, St. Louis, MO, United States) for 12 h at 37°C. Then, the mixture was centrifuged at 300 g for 5 min. The obtained cells and partially digested tissues were resuspended in complete medium for mesenchymal stem cells (Cyagen Biosciences Inc., Guangzhou, China) and cultured at 37°C in a humidified atmosphere containing 5% CO_2_. The culture medium was changed every 3 days, and cells were passaged on reaching 80–90% confluence. The NPSCs of second and third passage were used in following experiments.

### Cell Treatment

To mimic the *in vivo* compression environment, a stainless-steel pressure vessel was utilized following the protocols previously established by our group (Chen et al., 2017, 2018; Li et al., 2018a; Wang W. et al., 2018). NPSCs which were cultured in cell culture dishes or plates as monolayer were placed on the bracket in the pressure apparatus and exposed to 1.0 MPa pressure for different time periods. The pressure vessel was supplied with a small quantity of double distilled water at the bottom to maintain moisture and placed in an incubator at 37°C.

The HSP90 inhibitor BIIB021, RIPK1 inhibitor Necrostatin-1 (Nec-1), RIPK3 inhibitor GSK′872, MLKL inhibitor Necrosulfonamide (NSA), proteasome inhibitor MG132, and HSP70 inhibitor Ver155008 (Ver) were all purchased from Selleck Chemicals (Houston, TX, United States). For all experiments, NPSCs were pretreated with inhibitors for 2 h before compression treatment, while control groups were given isopyknic dimethylsulfoxide as vehicle control.

### Cell Viability Assay

Cell viability was examined using the cell counting kit 8 (CCK-8; Dojindo, Kyushu Island, Japan) following the manufacturer’s instructions. Briefly, NPSCs were seeded in 96-well culture plates at a density of 5 × 10^3^ cells per well and treated as above. At indicated time points, the culture medium was discarded, and 100 μL of DMEM/Ham’s F-12 (DMEM/F-12; Thermo Fisher Scientific, Waltham, MA, United States) and 10 μL of CCK-8 reagent were added to each well. Then, the absorbance was detected after incubation for 3 h at 37°C at a wavelength of 450 nm on a microplate reader (Biotek, Winooski, VT, United States).

### Lactate Dehydrogenase (LDH) Release Assay

Cells were seeded in 96-well culture plates at a density of 5 × 10^3^ cells per well and treated as above. The release of LDH from NPSCs to culture medium was examined using the LDH Cytotoxicity Assay Kit (Beyotime, Shanghai, China) according to manufacturer’s instructions. The absorbance was detected at a wavelength of 490 nm using a microplate reader (Biotek).

### Transmission Electron Microscopy (TEM)

The ultrastructure of NPSCs was observed using TEM following previously described methods (Chen et al., 2017, 2018). Briefly, after indicated treatments, cells were collected and pelleted by centrifugation at 1000 g for 15 min. The pellets were then fixed with 2.5% glutaraldehyde in PBS for 2 h and post-fixed with 1% osmium tetroxide for 2 h at room temperature. Next, the pellets were dehydrated in a graded series of ethanol and embedded in epon 812. Ultrathin sections were stained with uranyl acetate and lead citrate, and were examined utilizing the Tecnai G2 12 transmission electron microscope (FEI Company, Holland).

### Live and Dead Cell Staining

The membrane permeable probe Calcein-AM (Santa Cruz Biotechnology, lnc., Dallas, TX, United States) and propidium iodide (PI) (Nanjing Keygen Biotech, Nanjing, China) were used to label the live and dead cells, respectively. After indicated treatments, the NPSCs were washed twice with PBS. Then, the cells were incubated with Calcein-AM (2 μM) at 37°C for 20 min in the dark. After being gently rinsed twice with PBS, cells were further stained with PI according to manufacturer’s instructions. The live (green fluorescence) and dead (red fluorescence) cells were imaged by fluorescence microscopy (Olympus IX71, Japan).

### Transfection of siRNA

The siRNAs for HSP90α and HSP90β were published previously and synthesized by RiboBio Co (Guangzhou, China) (Shang and Tomasi, 2006). The target sequences of siRNAs were as follows: HSP90α, 5′-CCCAGUUGAUGUCAUUGAUCAUCAA-3′; HSP90β, 5′-GGCAGAGGAAGAGAAAGGUGAGAAA-3′. The negative control (NC) siRNA was also purchased from RiboBio Co. The transfection was performed utilizing the Lipofectamine 3000 (Thermo Fisher Scientific) according to manufacturer’s instructions, and the transfection efficacy was tested by real-time PCR (RT-PCR) and Western blot (WB).

### Immunofluorescence Staining

NPSCs which were seeded on glass coverslips were washed with PBS, and fixed by 4% paraformaldehyde for 15 min. Cells were then permeabilized with 0⋅5% Triton X-100 (Beyotime) for 15 min at room temperature (for staining of Tie2, the permeabilization was not performed), and blocked with goat serum albumin for 1 h. Next, samples were washed with PBS and incubated with the mixture of mouse anti-HSP90 antibody (1:200, Santa Cruz Biotechnology) and rabbit anti-phosphorylated MLKL (P-MLKL) antibody (Ser358, 1:200, Affinity Biosciences, OH, United States), the rabbit anti-HSP70 antibody (1:500, ABclonal, Wuhan, China), or rabbit anti-Tie2 antibody (1:100, Proteintech Group, Wuhan, China) at 4°C overnight, followed by incubation with fluorophore-conjugated secondary antibody (1:200, Proteintech Group). Finally, the nuclei were stained with 4′-6-diamidino-2-phenylindole (DAPI). Fluorescence images were observed using fluorescence microscope (Olympus IX71).

### Annexin V and PI Staining

The Annexin V-fluorescein isothiocyanate (FITC)/PI Apoptosis Detection Kit (Nanjing Keygen Biotech), and the Cell Apoptosis Detection Kit (PI) (Nanjing Keygen Biotech) were used to quantify the apoptotic and necrotic ratio of NPSCs, respectively. Briefly, after compression treatment, cells were harvested by trypsinization and washed twice with PBS. Then, the cells were stained according to manufacturer’s instructions. All samples were subsequently analyzed by flow cytometry (BD LSR II, Becton Dickinson).

### Terminal Deoxynucleotidyl Transferase Biotin-dUTP Nick end Labeling (TUNEL) Assay

TUNEL assays were performed using the One Step TUNEL Apoptosis Assay Kit (Beyotime) following manufacturer’s instructions. Briefly, cells were fixed with 4% formaldehyde for 30 min and permeabilized by 0.3% Triton X-100 for 5 min. Subsequently, the cells were incubated with TUNEL reaction mixture for 1 h at 37°C in the dark, counterstained with DAPI and observed under a fluorescence microscope (Olympus IX71). The cells with green fluorescence were defined as apoptotic cells.

### Evaluation of Mitochondrial Membrane Potential (MMP)

After compression treatment, NPSCs were stained with the fluorescent probe 5,5′,6,6′-tetrachloro-1,1′,3,3′-tetraethyl-benzimidazolylcarbocyanine iodide (JC-1, Nanjing Keygen Biotech) according to manufacturer’s instructions. Then, the stained cells were analyzed by flow cytometry (BD LSRII, Becton Dickinson) and observed using fluorescence microscope (Olympus IX71).

### ATP Production Assay

The ATP content was assessed using the Enhanced ATP Assay Kit (Beyotime). Briefly, after compression treatment, NPSCs were lysed with the ATP lysis buffer. Then, the supernatant was collected by centrifugation at 12,000 g for 5 min and reacted with the ATP detection working dilution. The luminescence activity was detected by luminescence spectrometry (EnSpire, United States). The ATP level was normalized to cellular protein concentration.

### Mitochondrial ROS (mtROS) Analysis

The mtROS was measured using MitoSOX Red (Thermo Fisher Scientific) staining according to manufacturer’s instructions. After compression treatment, the NPSCs were incubated with 5 μM MitoSOX Red for 10 min at 37°C. The NPSCs were then washed twice and counterstained with DAPI at 37°C for 10 min. Images were acquired using fluorescence microscope (Olympus IX71).

### Measurement of Cellular ROS

The intracellular ROS level was measured using the ROS Assay Kit (Beyotime) according to manufacturer’s instructions. The NPSCs were harvested and washed twice with PBS. Then, cells were incubated with 2′-7′-dihydrodichlorofluoroscein diacetate (DCFH-DA) in the dark at 37°C for 20 min. After being washed with DMEM/F-12 for three times, the fluorescence intensity was detected using flow cytometry (BD LSR II, Becton Dickinson). Intracellular ROS level in NPSCs was also observed under a fluorescence microscope (Olympus IX71).

### Malondialdehyde (MDA) Detection

The MDA concentration was determined using the Lipid Peroxidation MDA Assay Kit (Beyotime). NPSCs were washed with PBS, lysed with lysis buffer and centrifuged at 120,00 g for 10 min. Then, the supernatant was collected and reacted with the thiobarbituric acid. After centrifugation, the absorbance of supernatant was measured using spectrophotometer (EnSpire) at a wavelength of 532 nm. The MDA concentration was normalized to cellular protein concentration.

### WB Analysis

Total protein of cells was extracted using radioimmunoprecipitation assay buffer (Beyotime) containing the protease and phosphatase inhibitors. The protein concentration was determined utilizing the Enhanced BCA Protein Assay Kit (Beyotime). Equal amount of protein was electrophoresed in 8–12% sodium dodecyl sulfate-polyacrylamide gel and transferred onto polyvinylidene difluoride membranes (EMD Millipore, Billerica, MA, United States). After being blocked with 5% bovine serum albumin in Tris Buffered Saline with Tween 20 (TBST) buffer for 1 h at room temperature, the polyvinylidene difluoride membranes were incubated overnight at 4°C with primary antibodies against: HSP90 (1:500), RIPK1 (1:1000, Abcam, Cambridge, MA, United States), phosphorylated RIPK1 (P-RIPK1) (Ser166, 1:1000, Cell Signaling Technology, Danvers, MA, United States), RIPK3 (1:1000, Abcam), phosphorylated RIPK3 (P-RIPK3) (Ser227, 1:1000, Abcam), MLKL (1:1000, Abcam), P-MLKL (1:1000), Bcl-2 (1:1000, Abcam), Bax (1:500, Proteintech Group), Caspase-3 (1:500, Proteintech Group), poly ADP-ribose polymerase (PARP) (1:500, Proteintech Group), HSP70 (1:500), c-jun-N-terminal kinase (JNK) (1:1000, Cell Signaling Technology), phosphorylated JNK (P-JNK) (Thr183/Tyr185, 1:1000, Cell Signaling Technology), superoxide dismutase 2 (SOD2) (1:500, Proteintech Group), HSP90α (1:1000, ABclonal), HSP90β (1:500, ABclonal), GAPDH (1:2000, Affinity Biosciences). Subsequently, the membranes were washed three times with TBST and incubated with corresponding horseradish peroxidase-conjugated secondary antibodies for 1 h at room temperature. The protein bands were detected using enhanced chemiluminescence reagent system (Affinity Biosciences) following the manufacturer’s instructions.

### RT-PCR

Total RNA was extracted from NPSCs using the TRIzol reagent (Thermo Fisher Scientific) according to manufacturer’s instructions. Then, the concentration and purity of the total RNA were determined using the NanoDrop 2000. The isolated RNA was reverse-transcribed into corresponding cDNA using the reverse transcriptional kit (TaKaRa Bio, Tokyo, Japan). Quantitative RT-PCR was performed using the SYBR PrimeScript RT-PCR Kit (TaKaRa Bio) on the Step One Plus Real-Time PCR system (Thermo Fisher Scientific). *GAPDH* was used as the endogenous control, and relative mRNA expression levels of target genes were calculated using the 2^−ΔΔ*Ct*^ method. The primer sequences were designed and synthesized as follows: *HSP90AA1*, F: 5′-TCTCCACAGGGCTTGTTTTCC-3′, R: 5′-CA TTTTGGCACTAACTGTCATCC-3′; *HSP90AB1*, F: 5′-CCAG GCACTTCGGGACAAC-3′, R: 5′-TCAAACAGCAGCACCACC AG- 3′; *GAPDH*, F: 5′-AATCCCATCACCATCTTCCAG-3′, R: 5′-GAGCCCCAGCCTTCTCCAT-3′.

### Surgical Procedure of Animal Experiments

All animal procedures were performed under the approval of the animal experimentation committee of Huazhong University of Science and Technology. Twenty skeletally mature 12-week-old male Sprague-Dawley rats, ranging from 380 to 450 g, were obtained from the Experimental Animal Center of Huazhong University of Science and Technology. A previously reported rat tail model of disc degeneration induced by mechanical loading was employed in current study (Yurube et al., 2014; Yan et al., 2017). Rats were anesthetized by intraperitoneal injection of 50 mg/kg pentobarbital. The disc levels in rat tail were located by palpation on the coccygeal vertebrae. Then, rat tails were fixed with an Ilizarov-type apparatus. In brief, two cross 0.80 mm diameter Kirschner wires were inserted percutaneously into the 8th and 10th caudal vertebral bodies perpendicular to the tail’s axis, respectively. The Kirschner wires were subsequently attached to stainless steel rings which were connected longitudinally with four threaded rods. After instrumentation, axial force was generated utilizing four calibrated springs (0.50 N/mm) installed over each rod. In present study, the compressive stress of 1.3 MPa which was in accordance with the transient disc loading force produced by lifting a moderate weight in human lumbar spine was used to induce the disc degeneration of rat tail (Lotz and Chin, 2000). Rat tails with the compressive apparatus unloaded were regarded as the sham group. Following surgery, the caudal segments 8/9 and 9/10 were injected with vehicle in sham group, or randomly injected with BIIB021 (1000 nM/L) and vehicle in loaded group, respectively. To avoid disc degeneration induced by puncture, a micro-syringe attached to a 30-gauge needle (Hamilton Bonaduz AG, Bonaduz, Switzerland) was used, and the injection volume was 2 μL (Zou et al., 2013; Yurube et al., 2014). Following surgery, all animals were supplied with antibiotics and allowed unrestricted food, water and activity. The injections were conducted every 4 days. After 20 days, the rats were euthanized, and the discs were harvested, fixed in 4% paraformaldehyde, decalcified in 10% ethylenediaminetetraacetic acid and embedded in paraffin.

### Immunohistochemistry (IHC)

The paraffin-embedded tissue samples were cut into 4-μm thickness sections. The sections were then deparaffinized, rehydrated and incubated in 3% H_2_O_2_ for 20 min at room temperature to block the endogenous peroxidase activity. Antigen retrieval was performed by pressure cooking in 10 mmol/L citrate buffer, pH 6. Subsequently, the sections were blocked with 10% goat serum albumin for 30 min at room temperature, followed by incubation with rabbit anti-Tie2 polyclonal antibody (1:100), mouse anti-HSP90 antibody (1:50) at 4°C overnight. After being washed, the sections were labeled with horseradish peroxidase-linked goat anti-rabbit secondary antibody at 37°C for 1 h, and counterstained with hematoxylin.

### Statistical Analysis

All experiments were repeated at least three times, and data were presented as mean ± SD. Statistical analysis was performed using SPSS statistical software program 20⋅0 (IBM, Armonk, NY, United States). Differences between groups were analyzed by Student’s *t*-test or one-way analysis of variance (ANOVA) followed by Bonferroni *post hoc* test. *P* < 0⋅05 was considered statistically significant.

## Results

### Compression Induced Necrotic Cell Death of NPSCs

**FIGURE 1:**
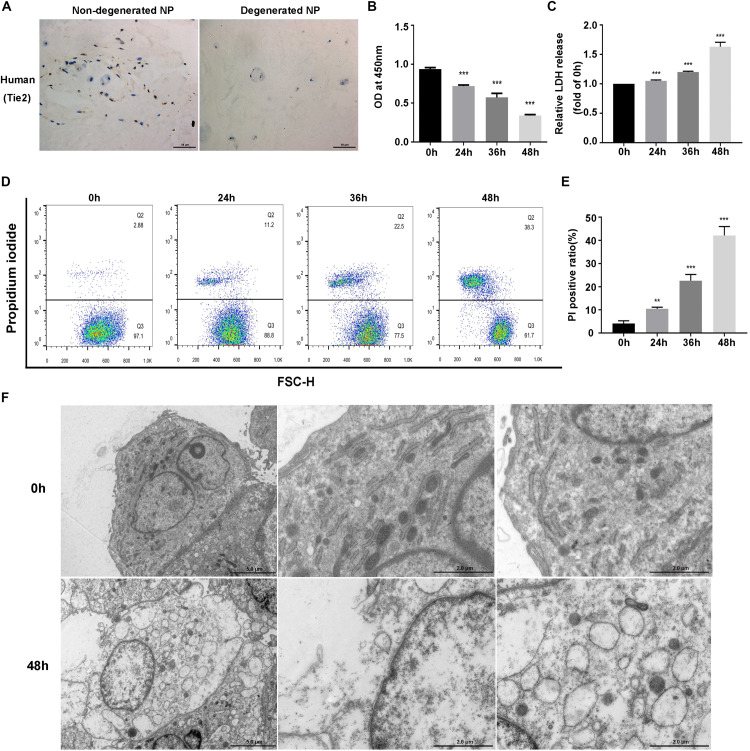
Compression induced necrotic cell death of NPSCs. **(A)** IHC staining of Tie2 for marking NPSCs in non-degenerated (37 years old, male, grade II) and degenerated (43 years old, male, grade IV) human NP tissues (original magnification: ×400). **(B)** Cell viability of NPSCs examined by CCK-8 assays. **(C)** The relative release of LDH at different time points. **(D)** Representative dot plots of PI staining obtained from flow cytometry analysis of NPSCs. **(E)** The statistical analysis of PI positive ratio of NPSCs. **(F)** The morphological ultrastructural appearance of NPSCs observed by TEM. The NPSCs exposed to 48 h of compression displayed necrotic morphological changes, such as severe vacuolation, swelling of organelles and disruption of the plasma membrane. The data were expressed as mean ± SD from at least three independent experiments, and they were analyzed by a two-tailed *t*-test or ANOVA. (**P < 0.01, ***P < 0.001 vs. 0 h).

We first detected the presence of NPSCs in human non-degenerated and degenerated NP tissues. Tie2, the specific surface marker for NPSCs, was used to label cells in tissue samples (Sakai et al., 2012; Tekari et al., 2016). In present study, we also used Tie2 to identify the purity of NPSCs (Supplementary Figure S1). The IHC showed that NPSCs existed in human non-degenerated and degenerated NP tissues. However, in degenerated NP tissues, the frequency of Tie2 positive cells declined dramatically, indicating the exhaustion of NPSCs (Figure 1A). We subsequently examined the influence of compression on viability of NPSCs. The CCK-8 assays demonstrated that cell viability of NPSCs was reduced by compression treatment in time-dependent manner (Figure 1B). Furthermore, prolonged compression treatment also led to increased LDH release (Figure 1C). We then determined whether compression induced necrosis of NPSCs. As expected, the flow cytometric analyses of PI staining showed that there was a marked increase in PI-positive cells in response to compression, indicating increased cellular necrosis (Figures 1D,E). In addition, we also utilized the TEM to observe the ultrastructure of NPSCs. As shown in Figure 1F, after exposure to compression for 48 h, the ultrastructure of cells was extensively damaged, as manifested by severe vacuolation, swelling of organelles and disruption of the plasma membrane. Taken together, above results suggested that compression could induce necrotic cell death of NPSCs.

### RIPK1/RIPK3/MLKL-Mediated Necroptosis Was Involved in Compression-Induced Death of NPSCs

**FIGURE 2:**
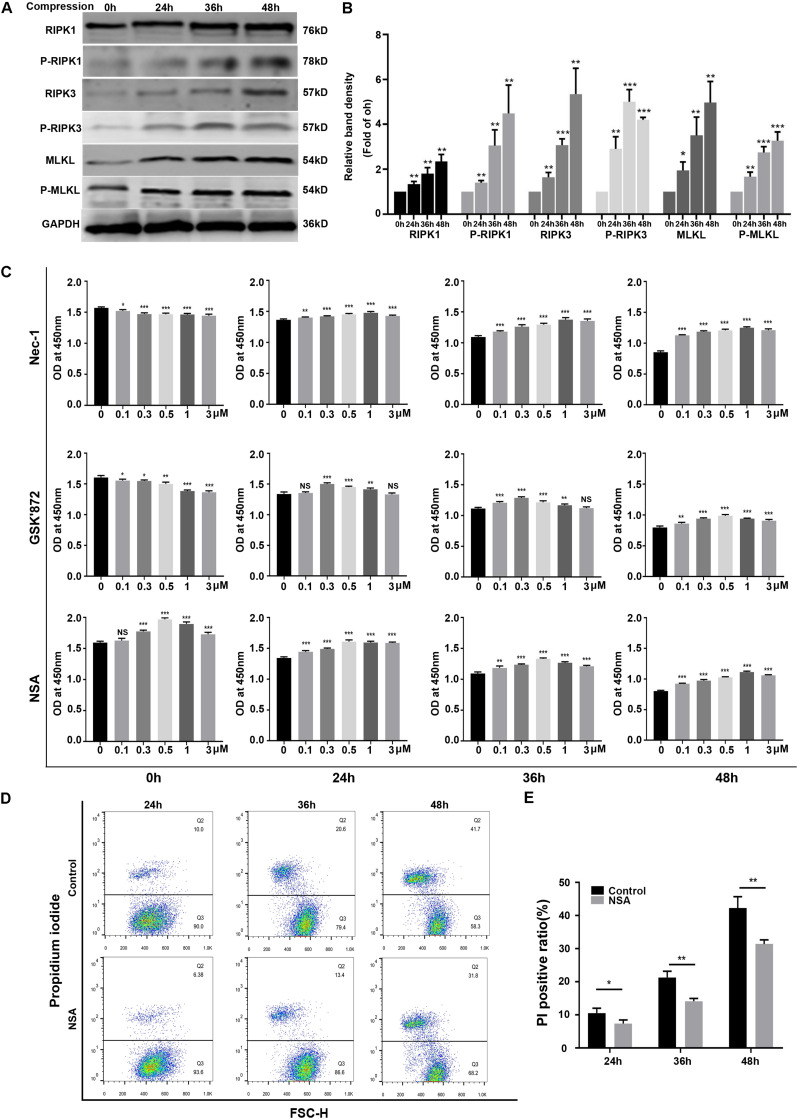
RIPK1/RIPK3/MLKL-mediated necroptosis was involved in compression-induced death of NPSCs. **(A)** Representative WB graphs of the expression of RIPK1, P-RIPK1, RIPK3, P-RIPK3, MLKL, and P-MLKL. **(B)** Quantitation of the expression levels of RIPK1, P-RIPK1, RIPK3, P-RIPK3, MLKL, and P-MLKL. **(C)** The effects of different concentrations of Nec-1, GSK′872 and NSA on cell viability of NPSCs exposed to 0, 24, 36, and 48 h compression measured by CCK-8 assays. **(D)** Representative dot plots of PI staining obtained from flow cytometry analysis of NPSCs. **(E)** The statistical analysis of PI positive ratio of NPSCs. The data were expressed as mean ± SD from at least three independent experiments, and they were analyzed by a two-tailed *t*-test. (*P < 0.05, **P < 0.01, ***P < 0.001 vs. 0 h, 0 μM or control, NS, not significant).

To investigate whether necroptosis was involved in compression-induced NPSCs death, we first examined the expression of key proteins of necroptosis signaling pathway. The WB results demonstrated that the expression levels of RIPK1, P-RIPK1, RIPK3, P-RIPK3, MLKL, and P-MLKL were all increased by compression treatment (Figures 2A,B). To further confirm the involvement of necroptosis in compression-induced NPSCs death, we used different concentrations of RIPK1 inhibitor Nec-1, RIPK3 inhibitor GSK′872 or MLKL inhibitor NSA to treat NPSCs, respectively. The results of CCK-8 assays showed that inhibiting RIPK1, RIPK3, or MLKL could also preserve the viability of NPSCs in response to compression (Figure 2C). In addition, pretreatment of NPSCs with NSA could also decrease the ratio of PI positive cells caused by compression (Figures 2D,E). Collectively, these results indicated that RIPK1/RIPK3/MLKL-mediated necroptosis was involved in compression-induced death of NPSCs.

### Inhibition of HSP90 Attenuated Compression-Induced NPSCs Death

**FIGURE 3:**
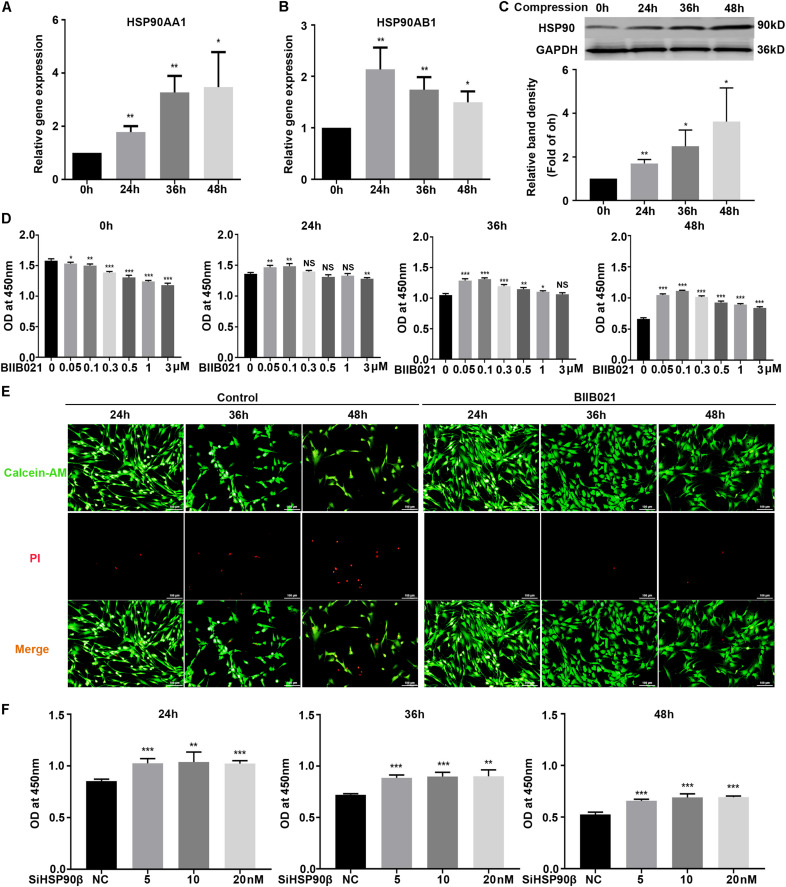
Inhibition of HSP90 attenuated compression-induced NPSCs death. **(A,B)** The expression levels of *HSP90AA1* **(A)** and *HSP90AB1* **(B)** measured by RT-PCR in human NPSCs. Data were normalized to *GAPDH*. **(C)** Representative WB graphs and quantitation of the expression level of HSP90. **(D)** The effects of different concentrations of BIIB021 on cell viability of NPSCs exposed to 0, 24, 36, and 48 h compression measured by CCK-8 assays. **(E)** Typical fluorescence photomicrograph of live/dead cell staining of NPSCs. Green fluorescent signaling (Calcien-AM) indicates live cells and red fluorescent signaling (PI) indicates dead cells (original magnification: ×200). **(F)** The effects of SiHSP90β on cell viability of NPSCs exposed to 24, 36, and 48 h compression measured by CCK-8 assays. The data were expressed as mean ± SD from at least three independent experiments, and they were analyzed by a two-tailed *t*-test. (*P < 0.05, **P < 0.01, ***P < 0.001 vs. 0 h, 0 μM or NC, NS, not significant).

To investigate the roles of HSP90 in compression-induced NPSCs death, we first detected the expression of HSP90 in human NP tissues. The IHC results demonstrated that HSP90 expression was elevated in degenerated NP tissues, indicating the involvement of HSP90 in the process of IVDD (Supplementary Figure S2). We further evaluated the expression levels of HSP90 in NPSCs in response to compression. The RT-PCR results demonstrated that the expression levels of *HSP90AA1* and *HSP90AB1* were elevated by compression treatment, which peaked at 48 h and 24 h, respectively (Figures 3A,B). In addition, the protein expression of HSP90 was also increased in a time-dependent manner (Figure 3C). We subsequently pretreated NPSCs with different concentrations of HSP90 inhibitor BIIB021. As shown in Figure 3D, pretreating NPSCs with BIIB021 attenuated the loss of viability caused by compression treatment. Since the cytoprotective effect was most prominent in concentration of 100 nM, therefore, the BIIB021 concentration of 100 nM was chosen for the following experiments. We then detected the influence of BIIB021 treatment on the expression of HSP90. The WB results showed that 100 nM of BIIB021 treatment could promote the expression of HSP90 in NPSCs under compression (Supplementary Figure S3). We further observed the survival of NPSCs using the live/dead cell staining. Consistently, live/dead cell staining displayed that the number of dead cells (red fluorescence) decreased, while the number of live cells (green fluorescence) increased in BIIB021 groups compared to that in control groups (Figure 3E).

To further verify the cytoprotective effects of inhibiting HSP90, HSP90 specific siRNAs were used. The transfection efficacy was validated by RT-PCR and WB, and the expression of HSP90 was dramatically downregulated at both protein and mRNA levels (Supplementary Figures S4A,B). Then, the CCK-8 assays were employed to examine the effects of HSP90 specific siRNAs on NPSCs viability. Interestingly, only when NPSCs were transfected with HSP90β siRNA could the loss of viability be attenuated (Figure 3F). In the meanwhile, transfection of NPSCs with HSP90α siRNA didn’t have significant influence on cell viability (Supplementary Figure S4C). Furthermore, co-transfection of NPSCs with HSP90β (10 nM) siRNA and different concentrations of HSP90α siRNAs also failed to rescue the loss of viability (Supplementary Figure S4D). These evidences demonstrated that HSP90 exerted pro-death roles in compression-induced NPSCs death, and inhibiting rather than silencing HSP90 could effectively attenuate compression-induced NPSCs death.

### Enhanced Expression of HSP70 Contributed to the Cytoprotective Effects of Inhibiting HSP90

**FIGURE 4:**
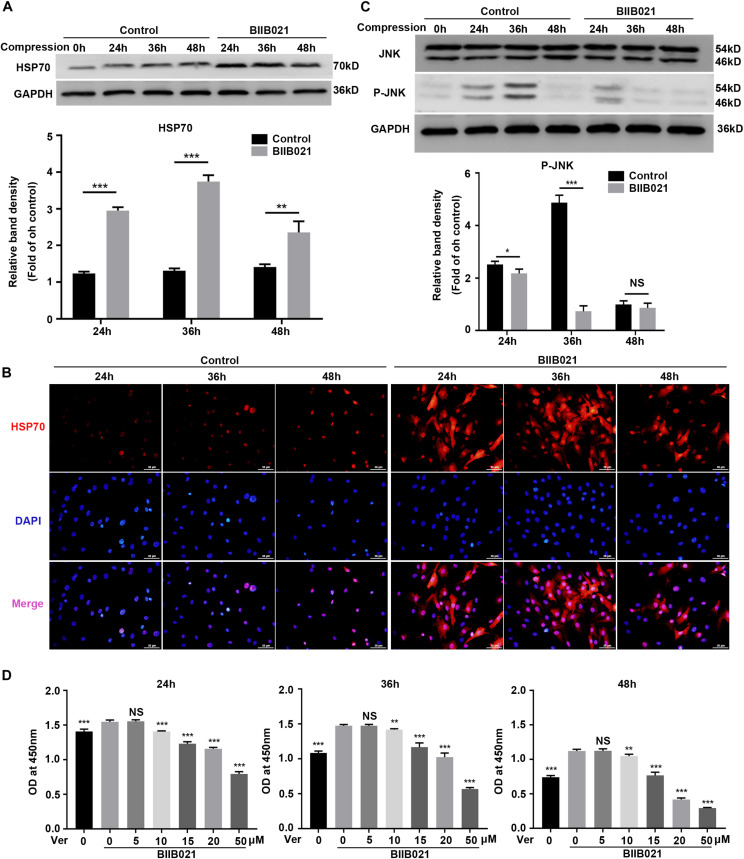
Enhanced expression of HSP70 contributed to the cytoprotective effects of inhibiting HSP90. **(A)** Representative WB graphs and quantitation of the expression levels of HSP70. **(B)** The representative fluorescence photomicrograph of HSP70 expression detected by immunofluorescence staining (original magnification: ×400). **(C)** Representative WB graphs and quantitation of the expression levels of JNK and P-JNK. **(D)** Ver partly reversed the protective effects of BIIB021 on NPSCs exposed to 24, 36, and 48 h compression measured by CCK-8 assay. The data were expressed as mean ± SD from at least three independent experiments, and they were analyzed by a two-tailed *t*-test. (*P < 0.05, **P < 0.01, ***P < 0.001 vs. control or Ver 0 nM+BIIB021 100 nM group, NS, not significant).

Previous studies have revealed that inhibition of HSP90 could result in increased HSP70 synthesis, which might be the reason for the protective effects of inhibiting HSP90 (Alani et al., 2014). Thus, we next evaluated the expression levels of HSP70 in NPSCs. As expected, the WB results showed that compression increased the expression of HSP70 protein in time-dependent manner, while the inhibition of HSP90 further promoted the expression of HSP70 (Figure 4A). Consistently, the immunofluorescence results displayed that inhibition of HSP90 notably enhanced the fluorescence intensity of HSP70 both in nuclei and cytoplasm (Figure 4B). HSP70 exerts its cytoprotective effects mainly by inhibiting JNK (Gabai et al., 1998). Therefore, we next examined the activity of JNK. The WB results showed that the expression of P-JNK was reduced by BIIB021 treatment at 24 and 36 h, indicating the decreased activity of JNK (Figure 4C). We further used HSP70 inhibitor Ver to treat NPSCs. The CCK-8 results showed that Ver treatment partially abolished the cytoprotective effects of HSP90 inhibitor (Figure 4D). Collectively, these results indicated that enhanced expression of HSP70 induced by HSP90 inhibitor contributed to the cytoprotective effects of inhibiting HSP90.

### Inhibiting HSP90 Suppressed Compression-Induced Necroptosis of NPSCs

**FIGURE 5:**
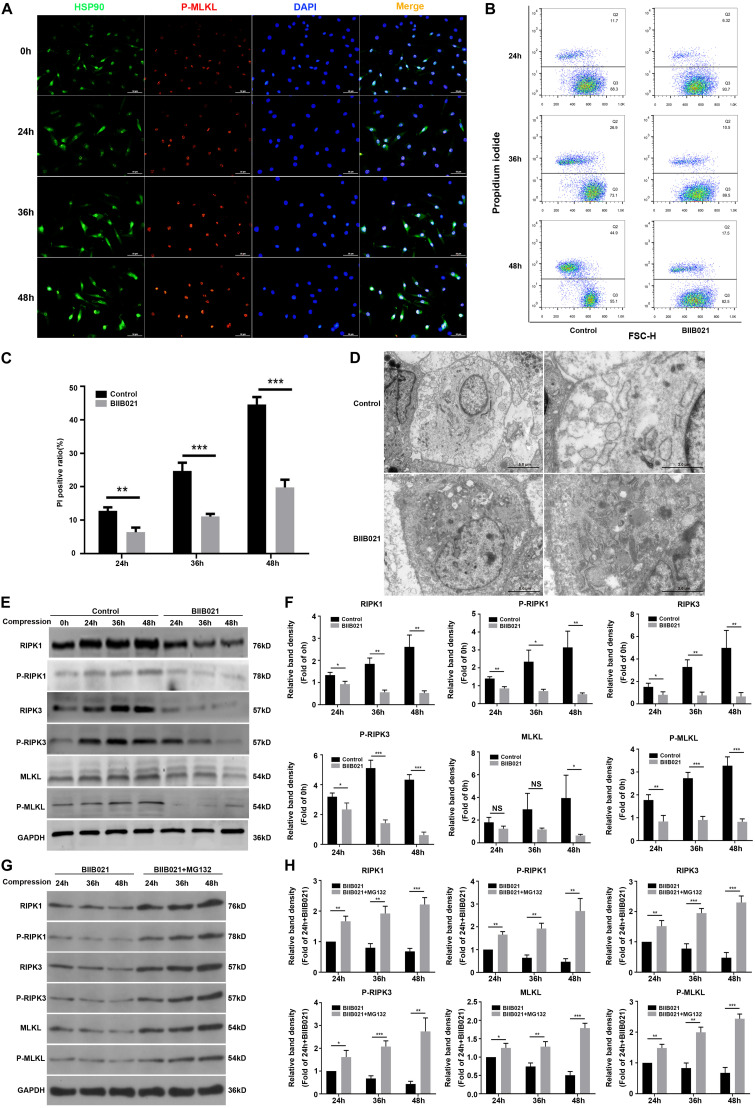
Inhibiting HSP90 suppressed compression-induced necroptosis of NPSCs. **(A)** The representative fluorescence photomicrograph of HSP90 and P-MLKL expression detected by immunofluorescence staining (original magnification: ×400). **(B)** Representative dot plots of PI staining obtained from flow cytometry analysis of NPSCs. **(C)** The statistical analysis of PI positive ratio of NPSCs. **(D)** The morphological ultrastructural appearance of NPSCs exposed to 48 h compression observed by TEM. **(E,F)** Representative WB graphs and quantitation of the expression levels of RIPK1, P-RIPK1, RIPK3, P-RIPK3, MLKL, and P-MLKL. **(G,H)** Representative WB graphs and quantitation of the expression levels of RIPK1, P-RIPK1, RIPK3, P-RIPK3, MLKL, and P-MLKL. The data were expressed as mean ± SD from three independent experiments, and they were analyzed by a two-tailed *t*-test. [*P < 0.05, **P < 0.01, ***P < 0.001 vs. control or BIIB021 **(G,H)**].

HSP90 is recently found to modulate the stability and function of RIPK1, RIPK3, and MLKL, indicating the regulatory effects of HSP90 on necroptosis (Yang and He, 2016). Using immunofluorescence, we observed that compression could elevate the expression of HSP90 and P-MLKL simultaneously (Figure 5A). More interestingly, we found that in cells which expressed higher levels of P-MLKL, the expression of HSP90 was also stronger, indicating that HSP90 might be associated with compression-induced necroptosis of NPSCs (Figure 5A). Therefore, we subsequently examined whether inhibiting HSP90 suppressed compression-induced necroptosis of NPSCs. The flow cytometric analyses showed that the proportion of PI positive cells was significantly decreased by BIIB021 treatment, indicating alleviated cellular necrosis (Figures 5B,C). The TEM results further confirmed that the necrotic ultrastructure features, such as the swelling of organelles and disruption of the plasma membrane, were alleviated by BIIB021 treatment (Figure 5D). Furthermore, we found that the expression of RIPK1, P-RIPK1, RIPK3, P-RIPK3, and P-MLKL were all dramatically inhibited by HSP90 inhibitor (Figures 5E,F). However, the inhibitory effect of BIIB021 on MLKL was significant only in 48 h group (Figures 5E,F). Then, we used HSP90 specific siRNAs to further verify the regulatory effects of HSP90 on necroptosis. Consistently, downregulation of HSP90 could also reduce the expression of RIPK1, P-RIPK1, RIPK3, P-RIPK3, MLKL, and P-MLKL (Supplementary Figures S5A,B). Previous studies have reported that HSP90 regulates necroptosis mainly by modulating the stability and function of RIPK1, RIPK3, and MLKL via ubiquitin–proteasome pathway (Yang and He, 2016). We then used proteasome inhibitor MG132 to explore whether HSP90 regulated the degradation of above proteins in NPSCs under compression. The WB results demonstrated that treatment of NPSCs with MG132 blocked the decline in the expression of RIPK1, P-RIPK1, RIPK3, P-RIPK3, MLKL, and P-MLKL induced by BIIB021 (Figures 5G,H). Collectively, these results indicated that inhibiting HSP90 could effectively suppress compression-induced necroptosis of NPSCs.

### Inhibiting HSP90 Attenuated Compression-Induced Mitochondrial Dysfunction of NPSCs

**FIGURE 6:**
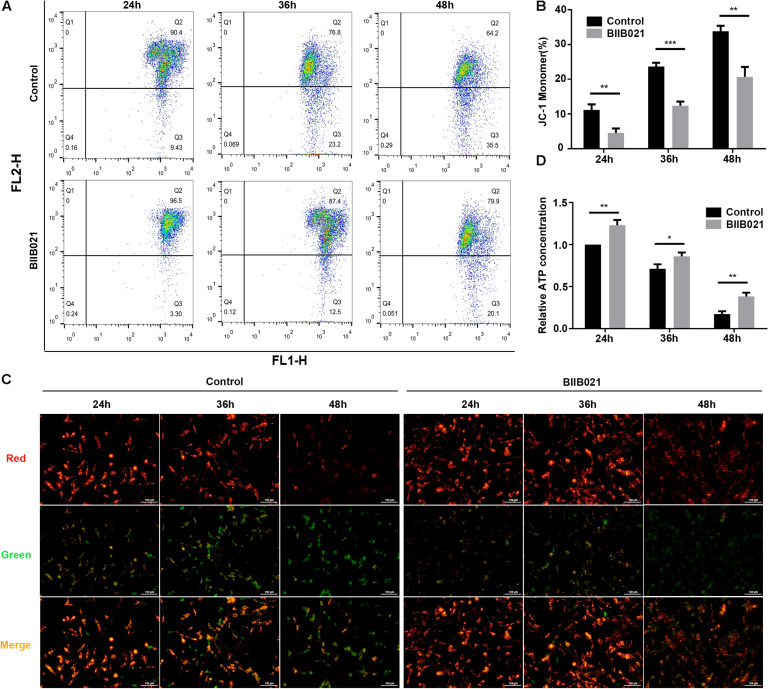
Inhibiting HSP90 attenuated compression-induced mitochondrial dysfunction of NPSCs. **(A)** Representative dot plots of JC-1 staining obtained from flow cytometry analysis of NPSCs for detecting the MMP. **(B)** The statistical analysis of MMP which was expressed as the ratio of JC-1 monomer. **(C)** Typical fluorescence photomicrograph of JC-1 staining in NPSCs (original magnification: ×200). **(D)** The protective effects of BIIB021 on compression-induced ATP depletion of NPSCs. The data were expressed as mean ± SD from three independent experiments, and they were analyzed by a two-tailed *t*-test. (*P < 0.05, **P < 0.01, ***P < 0.001 vs. control).

Mitochondrial dysfunction and ROS are critical in the execution of necroptosis (Marshall and Baines, 2014). Thus, to further confirm the inhibition of necroptosis by BIIB021, we investigated the effects of inhibiting HSP90 on mitochondria. Loss of MMP is regarded as the indicator of mitochondrial dysfunction. The flow cytometric analyses of JC-1 staining showed that inhibiting HSP90 notably alleviated the loss of MMP, as indicated by decreased ratio of JC-1 monomer in BIIB021 groups (Figures 6A,B). Additionally, the fluorescence imaging of JC-1 staining also confirmed the rescue effects of inhibiting HSP90 on MMP loss, as demonstrated by increased red fluorescence and decreased green fluorescence (Figure 6C). Further, we evaluated the intracellular ATP level of NPSCs. Our results showed that compression caused time-dependent decline of intracellular ATP, while the treatment of BIIB021 attenuated the depletion of intracellular ATP in NPSCs (Figure 6D). These data indicated that inhibiting HSP90 attenuated compression-induced mitochondrial dysfunction of NPSCs.

### Inhibiting HSP90 Alleviated Compression-Induced Oxidative Stress of NPSCs

**FIGURE 7:**
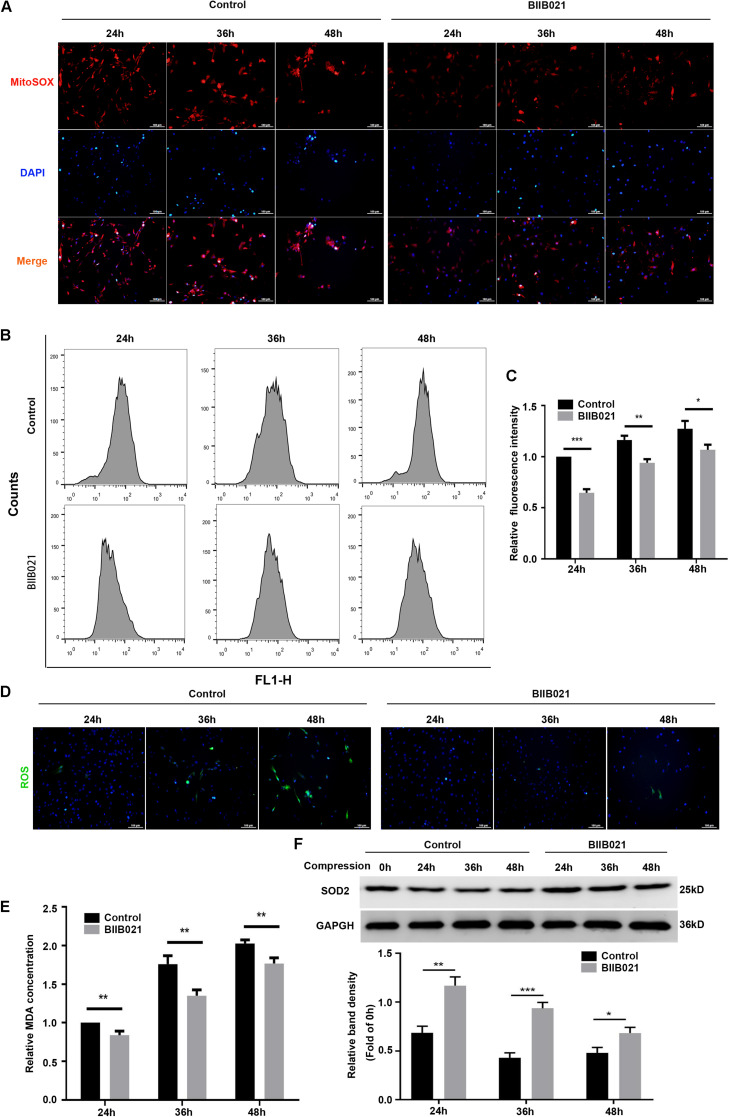
Inhibiting HSP90 alleviated compression-induced oxidative stress of NPSCs. **(A)** Typical fluorescence photomicrograph of mtROS in NPSCs probed by MitoSOX red (original magnification: ×200). **(B)** Representative dot plots of DCFH-DA staining obtained from flow cytometry analysis of NPSCs for detecting cellular ROS. **(C)** The statistical analysis of cellular ROS. **(D)** Typical fluorescence photomicrograph of cellular ROS in NPSCs (original magnification: ×200). **(E)** The relative content of intracellular MDA. **(F)** Representative WB graphs and quantitation of the expression levels of SOD2. The data were expressed as mean ± SD from three independent experiments, and they were analyzed by a two-tailed *t*-test. (*P < 0.05, **P < 0.01, ***P < 0.001 vs. control).

We first utilized the MitoSOX Red to evaluate the mtROS levels. As demonstrated by fluorescence images, an increasing production of mtROS was observed in compression-treated NPSCs, while BIIB021 treatment reduced the mtROS level (Figure 7A). Furthermore, we also used the DCFH-DA probe to label the intracellular total ROS. As indicated by flow cytometric analyses, inhibiting HSP90 could also largely attenuate the level of intracellular total ROS (Figures 7B,C). In addition, the fluorescence images also demonstrated the same tendency (Figure 7D). MDA is an indicator of lipid peroxidation, and SOD2 is an antioxidant enzyme. Corresponding to above results, we also found that inhibiting HSP90 decreased the MDA content and promoted the expression of SOD2 in NPSCs (Figures 7E,F). These results suggested that inhibiting HSP90 could efficiently alleviate compression-induced oxidative stress of NPSCs.

### Inhibiting HSP90 Protected NPSCs From Compression-Induced Apoptosis

**FIGURE 8:**
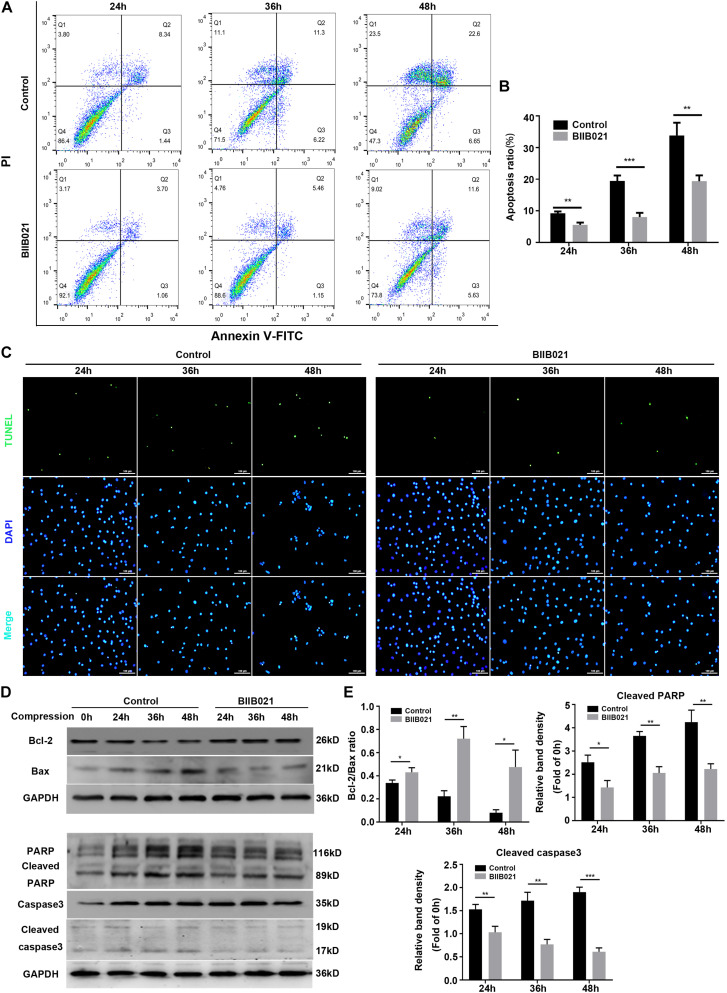
Inhibiting HSP90 protected NPSCs from compression-induced apoptosis. **(A)** Representative dot plots of Annexin V-FITC/PI staining obtained from flow cytometry analysis of NPSCs. The Annexin + /PI- and Annexin + /PI+ represent apoptotic cells. **(B)** The statistical analysis of apoptosis ratio of NPSCs. **(C)** Typical fluorescence photomicrograph of TUNEL staining of NPSCs (original magnification: ×200). **(D)** Representative WB graphs of the expression of Bcl-2, Bax, PARP, cleaved PARP, caspase3, and cleaved caspase3. **(E)** Quantitation of the ratio of Bcl-2/Bax and the expression levels of cleaved PARP, and cleaved caspase3. The data were expressed as mean ± SD from three independent experiments, and they were analyzed by a two-tailed *t*-test. (*P < 0.05, **P < 0.01, ***P < 0.001 vs. control, NS, not significant).

Since mitochondrial dysfunctional and ROS could trigger cell apoptosis through mitochondrial apoptosis pathway, we then determined whether HSP90 regulated compression-induced apoptosis of NPSCs (Feng et al., 2017). The flow cytometric analyses illustrated that inhibition of HSP90 alleviated compression-induced apoptosis of NPSCs in comparison with control groups (Figures 8A,B). In addition, the proportion of TUNEL-positive cells (green fluorescence) was also decreased in the presence of BIIB021, which intuitively confirmed the inhibitory effects of inhibiting HSP90 on apoptosis of NPSCs (Figure 8C). To further verify HSP90 inhibition-mediated blockage of apoptosis, we evaluated the expression levels of apoptosis-related proteins. The WB results demonstrated that inhibition of HSP90 could alleviate the decrease of Bcl-2/Bax ratio, and block the up-regulation of pro-apoptotic factors cleaved-caspase 3 and cleaved-PARP (Figures 8D,E). These multiple lines of evidences implied that besides necroptosis, inhibiting HSP90 could also attenuate compression-induced apoptosis of NPSCs.

### Inhibiting HSP90 Attenuated the Exhaustion of NPSCs *in vivo*

**FIGURE 9:**
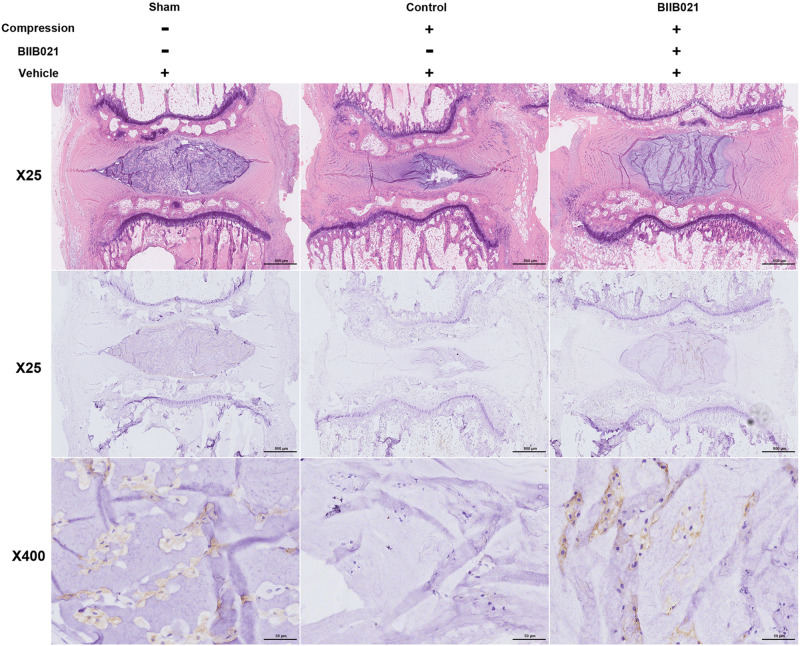
Inhibiting HSP90 attenuated the exhaustion of NPSCs *in vivo.* Hematoxylin and eosin staining (original magnification: ×25) and the IHC staining of Tie2 (original magnification: ×25 and 400) for labeling endogenous NPSCs of IVDs.

The aforementioned results demonstrated that inhibiting HSP90 could protect NPSCs from compression-induced death *in vitro.* To further explore whether inhibition of HSP90 exerted cytoprotective effects *in vivo*, a rat tail model of disc degeneration induced by mechanical loading was employed. As shown in Figure 9, the HE staining illustrated the degeneration of IVD in compression-treated group. Furthermore, as demonstrated by IHC, the frequency of Tie2-positive cells in NP tissue was decreased in compression-loaded groups compared to that in sham-operated group, indicating the exhaustion of NPSCs *in vivo* (Figure 9). Furthermore, compared to control group, the decline in the frequency of Tie2-positive cells in NP tissue was attenuated in BIIB021-injected group, which indicated the inhibition of NPSCs exhaustion (Figure 9). Together, these results suggested that compression could trigger the exhaustion of NPSCs, and inhibiting HSP90 could attenuate the exhaustion of NPSCs *in vivo*.

## Discussion

**FIGURE 10:**
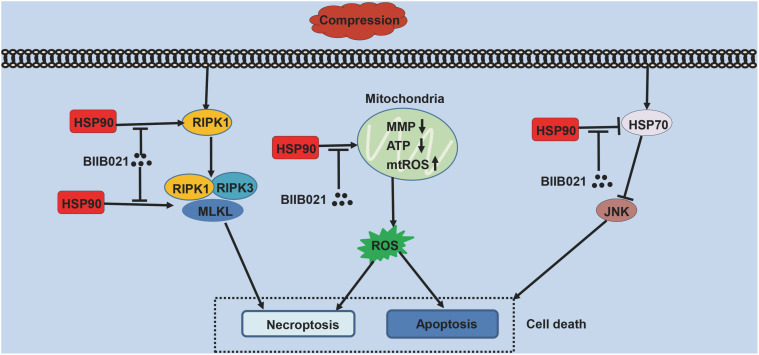
The schematic representation of the effects of HSP90 on compression-induced NPSCs death.

NPSCs provide novel prospects for the regeneration of degenerated IVD (Hu and He, 2018). However, with aging and degeneration, the frequency of NPSCs markedly decreases, indicating the exhaustion of NPSCs (Sakai et al., 2012). The precise mechanisms underlying the exhaustion of NPSCs have not yet been fully elucidated. As shown in Figure 10, in current study, we identified that compression could trigger RIPK1/RIPK3/MLKL-mediated necroptosis of NPSCs. Furthermore, we found that elevated expression of HSP90 was involved in compression-induced NPSCs death, and inhibiting HSP90 could dramatically attenuate compression-induced necroptosis of NPSCs via regulating the expression and activity of RIPK1/RIPK3/MLKL, and alleviating the mitochondrial dysfunction and oxidative stress. Besides necroptosis, compression-induced apoptosis of NPSCs was also attenuated by HSP90 inhibition. In addition, we found that enhanced expression of HSP70 also contributed to the cytoprotective effects of inhibiting HSP90. More encouragingly, our results demonstrated that inhibiting HSP90 could also mitigate the exhaustion of NPSCs *in vivo*. To our knowledge, this is the first study to identify the implication of necroptosis as well as the roles of HSP90 in compression-induced NPSCs death.

Cell death is the common characteristic for the pathology of various human diseases (Zhou and Yuan, 2014). Our group has demonstrated that compression could induce the apoptosis of NPSCs, which could partially explain the exhaustion of NPSCs (Li et al., 2018a). In present study, we found that compression could also trigger the necrotic cell death of NPSCs, as indicated by increased PI positive cells and the necrotic morphological features revealed by TEM. These results are in accordance with previous discovery which shows that the necrotic cells gradually increase with aging, and even 80% of total cells are necrotic in elderly people (Buckwalter, 1995). Necrosis is traditionally regarded as unregulated accidental cell death process. Recently, accumulating evidence reveals that a subset of necrosis, referred to as necroptosis, could occur in controlled and regulated manner (Hu et al., 2018). Our previous study proves that compression could induce RIPK1/RIPK3/MLKL-mediated necroptosis of NP cells (Chen et al., 2017). For NPSCs, our data showed that the expression levels and activity of RIPK1, RIPK3, and MLKL were increased by compression treatment. In addition, all of the Nec-1, GSK′872 and NSA could attenuate compression-induced viability loss of NPSCs. The flow cytometric analyses further confirmed that NSA could decrease the proportion of PI positive cells. Altogether, these results indicate that RIPK1/RIPK3/MLKL-mediated necroptosis is involved in compression-induced NPSCs death.

HSP90 is an abundantly expressed molecular chaperone, which plays important roles in many cellular processes (Jackson, 2013). Many kinds of adverse stimuli, such as the mechanical loading, oxidative stress and oxygen-glucose deprivation, could induce the expression of HSP90 (Siebelt et al., 2013; Chooi and Chan, 2016; Liu Q. et al., 2016; Wang Z. et al., 2018). We also detected that compression could increase the expression of HSP90 at both protein and mRNA levels in NPSCs. Our findings are consistent with former study which demonstrates that mechanical loading could upregulate the expression of HSP90 in NP cells encapsulated in 3D collagen constructs (Chooi and Chan, 2016). We then determined the effects of HSP90 on compression-induced NPSCs death. Our results demonstrated that inhibiting HSP90 with BIIB021 dramatically suppressed the death of NPSCs, indicating the pro-death role of HSP90 in compression-induced NPSCs death. Our findings are consistent with some former studies which show that HSP90 exerts pro-death functions in some pathological processes, such as traumatic brain injury and eye degeneration (Hooven et al., 2004; Ma et al., 2015). Furthermore, the cytoprotective effects of inhibiting HSP90 have also been proven in plenty of studies (Alani et al., 2014; Ma et al., 2015; Yin et al., 2017). However, we must notice that there are also studies supporting the pro-survival roles of HSP90 (Arya et al., 2007). We speculated that the discrepant effects of HSP90 on cell death might be due to different activity of the pro-survival and pro-death signaling pathways. HSP90 could interact with a wide spectrum of client proteins, which indicates that HSP90 participates in the regulation of numerous signaling pathways, including both the pro-survival and pro-death signaling pathways (Pearl and Prodromou, 2000). Thus, the different activity of the pro-survival and pro-death signaling pathways caused by HSP90 under different circumstance and cell types might result in different cell fate. However, this hypothesis needs to be verified in further studies. In present study, we used HSP90α and HSP90β specific siRNAs to further validate the protective effects of inhibiting HSP90. However, only the transfection of HSP90β siRNA could improve the viability of NPSCs, while the transfection of HSP90α siRNA or simultaneous transfection of both the HSP90α and HSP90β siRNAs had no influence on cell viability. Our findings imply that HSP90α and HSP90β might have different effects on NPSCs death, which needs further exploration.

In present study, we found that compression-induced necroptosis of NPSCs was dramatically attenuated by HSP90 inhibitor. We observed that inhibition of HSP90 decreased PI positive cells and alleviated necrotic ultrastructure features of NPSCs. We also found that the expression of core necroptosis regulators RIPK1, P-RIPK1, RIPK3, P-RIPK3 and P-MLKL were all dramatically inhibited by HSP90 inhibitor or HSP90 specific siRNAs. However, the inhibitory effect of BIIB021 on MLKL was significant only in 48 h group, which might be because that HSP90 mainly modulates the function of MLKL rather than simply controls its stability (Yang and He, 2016). Previous studies have revealed that HSP90 regulates necroptosis by modulating the stability and function of RIPK1, RIPK3, and MLKL via ubiquitin–proteasome pathway (Yang and He, 2016). We also observed that MG132 blocked the decline in the expression of core necroptosis regulators induced by BIIB021, which indicated that HSP90 regulated compression-induced necroptosis of NPSCs via modulating the stability of core necroptosis regulators. The regulatory effects of HSP90 on necroptosis are just recently established, and much of our knowledge comes from models based on the stimulation of tumor necrosis factor (Yang and He, 2016). Only (Wang Z. et al., 2018) have manifested the regulatory effects of HSP90 on necroptosis in the background of specific disease. Thus, our results provide novel evidence for the regulatory effects of HSP90 on necroptosis.

In addition to regulating the expression and activity of RIPK1, RIPK3, and MLKL, we found that inhibiting HSP90 could also alleviate the mitochondrial dysfunction and oxidative stress of NPSCs. Our results demonstrated that inhibiting HSP90 could mitigate the loss of MMP and the depletion of ATP. Moreover, inhibition of HSP90 reduced compression-induced production of mtROS, cellular total ROS and MDA, and promoted the expression of SOD2. Our results are consistent with former study which shows that silence of HSP90 could alleviate 6-hydroxydopamine-induced oxidative stress of PC12 cells via upregulation of NRF-2 (Alani et al., 2014). In addition, it is reported that inhibiting HSP90 could overcome hydrogen peroxide-induced death of neural stem cells, which also implies the anti-oxidative stress role of inhibiting HSP90 (Liu Q. et al., 2016). Mitochondrial dysfunction and ROS are critical in the execution of necroptosis (Marshall and Baines, 2014). Our group has demonstrated that mitochondrial dysfunction and oxidative stress contribute greatly to compression-induced NP cells necroptosis (Chen et al., 2018). Thus, alleviated mitochondrial dysfunction and oxidative stress could further confirm the inhibitory effect of HSP90 inhibitors on necroptosis. Mitochondrial dysfunction and ROS could also trigger cell apoptosis. Consistently, we observed that apoptosis of NPSCs induced by compression was alleviated by BIIB021 treatment. Consistent with our results, many studies have shown that HSP90 could exert pro-apoptotic effects in some diseases, such as traumatic brain injury, neurodegenerative disease and eye degeneration (Hooven et al., 2004; Alani et al., 2014; Ma et al., 2015). Furthermore, the inhibition of apoptosis by HSP90 inhibitors or siRNAs has also been established in many studies (Wang et al., 2011; Alani et al., 2014; Ma et al., 2015; Yin et al., 2017). In a word, the above results demonstrate that the apoptosis and necroptosis of NPSCs induced by compression could be simultaneously suppressed by HSP90 inhibitor. It is widely accepted that inhibiting apoptosis could enhance the necroptosis. Thus, simultaneous inhibition of apoptosis and necroptosis might be more efficient in protecting cells from death, and this concept has been proven in our previous study conducted in NP cells (Chen et al., 2018). Therefore, inhibiting HSP90 might be a promising potent strategy for preventing cells from death.

The induction of HSP70 by HSP90 inhibitors, which is associated with the activation of heat shock factor 1, has been documented in many studies (Siebelt et al., 2013; Alani et al., 2014; Kim et al., 2015). We also observed that inhibition of HSP90 notably augmented the expression of HSP70 in NPSCs. HSP70 is a ubiquitous molecular chaperone, which could regulate the intrinsic and extrinsic apoptotic pathways, thus protecting cells against various cellular stresses (Kumar et al., 2016). Besides apoptosis, some recent studies propose that HSP70 could also inhibit necroptosis via suppressing autophagy or RIPK1 activity (Liu X. et al., 2016; Srinivasan et al., 2018). Thus, enhanced expression of HSP70 is generally considered to be responsible for the cytoprotective effects of inhibiting HSP90. Consistently, we found that HSP70 inhibitor Ver could impair the cytoprotective effects of inhibiting HSP90. Furthermore, HSP70 is known to repress the activation of JNK (Gabai et al., 1998). Herein, we identified that the activation of JNK was inhibited by BIIB021 treatment. There are studies manifesting that activated JNK is extensively involved in apoptosis and programmed necrosis, such as necroptosis, pyroptosis and ferroptosis (Dhanasekaran and Reddy, 2017). Thus, in line with previous studies, above evidences strongly support that elevated expression of HSP70 contributes greatly to the cytoprotective effects of BIIB021 in NPSCs.

We also verified the cytoprotective effects of inhibiting HSP90 *in vivo* using a rat tail model of disc degeneration induced by mechanical loading. The *in vivo* results showed that compression treatment decreased the frequency of Tie2 positive cells, which further confirmed that compression was responsible for the exhaustion of NPSCs. BIIB021 treatment partially prevented the decline in the frequency of Tie2 positive cells, indicating the inhibition of NPSCs exhaustion. Our results are consistent with some previous studies which demonstrate that inhibiting HSP90 could protect the neural progenitor cells and intestinal stem cell niche (Wang et al., 2011; Joly et al., 2016). Currently, there are many HSP90 inhibitors being tested in clinical trials for antitumor therapy (Dickson et al., 2013; Muhammad Wasif et al., 2014; Piotrowska et al., 2018). Except for cancers, HSP90 inhibitors are also promising in treating osteoarthritis, traumatic brain injury, neurodegenerative disease et al. (Siebelt et al., 2013; Alani et al., 2014; Ma et al., 2015). Although some HSP90 inhibitors are well-tolerated and effective in treating cancers, we must notice that HSP90 inhibitors-associated dose-limiting toxicities are not accepted in treating IVDD. In present research, we proved that local administration of BIIB021 was effective in attenuating compression-induced exhaustion of NPSCs, which provides potential strategy for attenuating systemic side effects of HSP90 inhibitors. Thus, it might be possible for the translation of our results into the clinical therapy for IVDD.

## Conclusion

In conclusion, our results prove that RIPK1/RIPK3/MLKL-mediated necroptosis participates in compression-induced NPSCs death. Furthermore, we demonstrate that HSP90 plays pivotal roles in compression-induced NPSCs death. Inhibiting HSP90 could dramatically reduce the death of NPSCs via suppressing the cell apoptosis and necroptosis. Furthermore, our study might provide promising strategy for preventing the exhaustion of NPSCs, thus delaying or even reversing the degeneration of IVD.

## Data Availability Statement

The raw data supporting the conclusions of this article will be made available by the authors, without undue reservation.

## Ethics Statement

The studies involving human participants were reviewed and approved by The Medical Ethics Committee of the Tongji Medical College, Huazhong University of Science and Technology. The patients/participants provided their written informed consent to participate in this study. The animal study was reviewed and approved by The Animal Experimentation Committee of the Huazhong University of Science and Technology.

## Author Contributions

BH, SZ, and ZS designed the study and experiments. BH, SZ, WL, PW, XL, and DS conducted the experiments. BH, SZ, SC, BW, and YW drafted and edited the manuscript. KM, BW, and ZS supervised the experiments. All authors approved the final version of the manuscript.

## Conflict of Interest

The authors declare that the research was conducted in the absence of any commercial or financial relationships that could be construed as a potential conflict of interest.
