# Supplementary material for: Inhibiting Heat Shock Protein 90 Protects Nucleus Pulposus-Derived Stem/Progenitor Cells From Compression-Induced Necroptosis and Apoptosis
Source: Front Cell Dev Biol. 2020 Aug 7;8:685. doi: 10.3389/fcell.2020.00685 (PMC7427414; doi:10.3389/fcell.2020.00685)
Supplement: Supplementary file 1 [file Image_1.PDF]

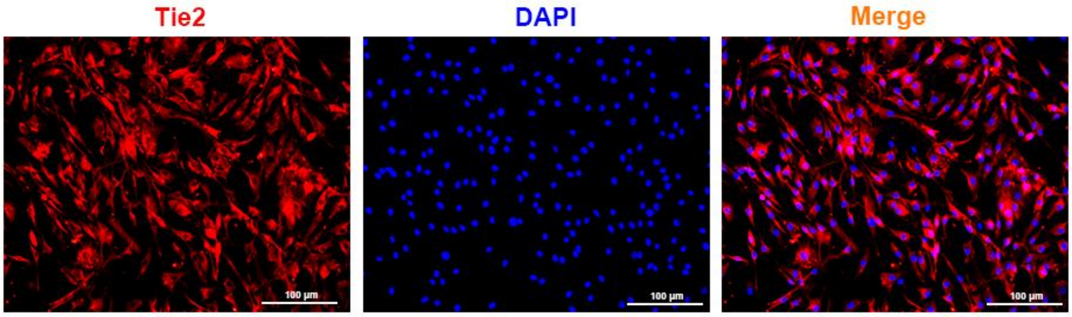

Supplementary Figure 1. The purity of NPSCs identified by Tie2 staining. The images show the typical fluorescence photomicrograph of Tie2 staining of NPSCs (original magnification: ×200). The rate of Tie2-positive cells was 95.91±1.425%.

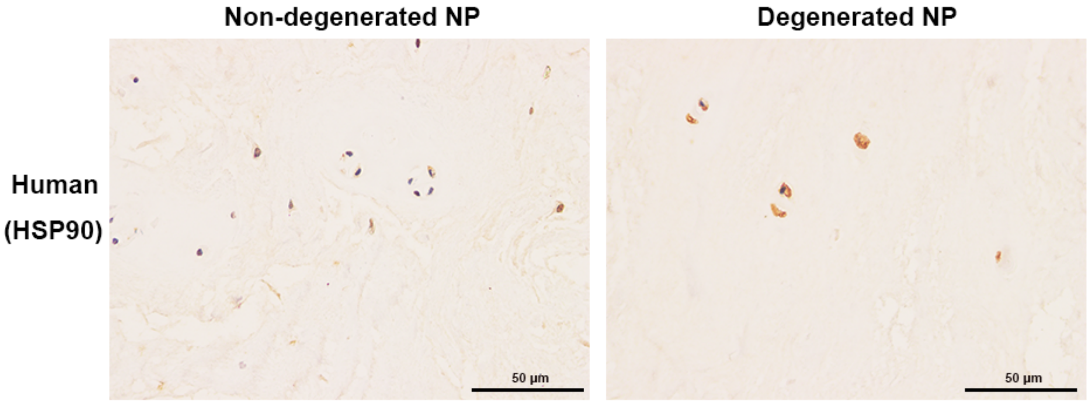

Supplementary Figure 2. IHC staining of HSP90 in non-degenerated (37 years old, male, grade II) and degenerated (43 years old, male, grade IV) human NP tissues (original magnification: ×400).

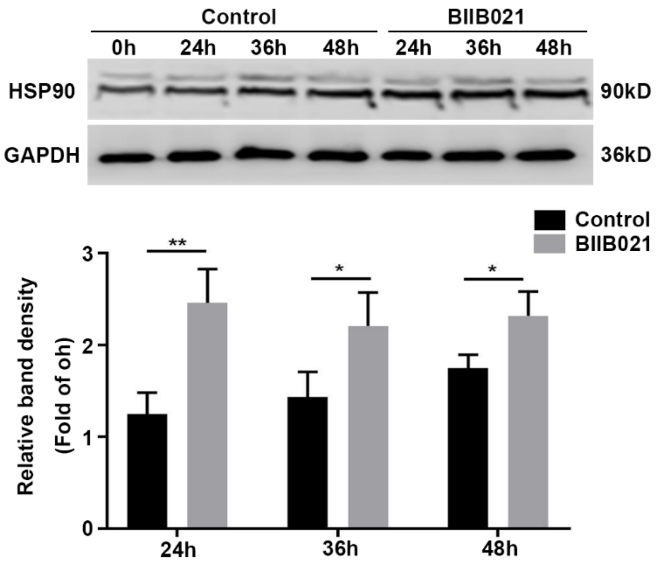

Supplementary Figure 3. Representative WB graphs and quantitation of the expression levels of HSP90. The data were expressed as mean ± SD from three independent experiments, and they were analyzed by a two-tailed t-test. (*P< 0.05, **P< 0.01 vs. control).

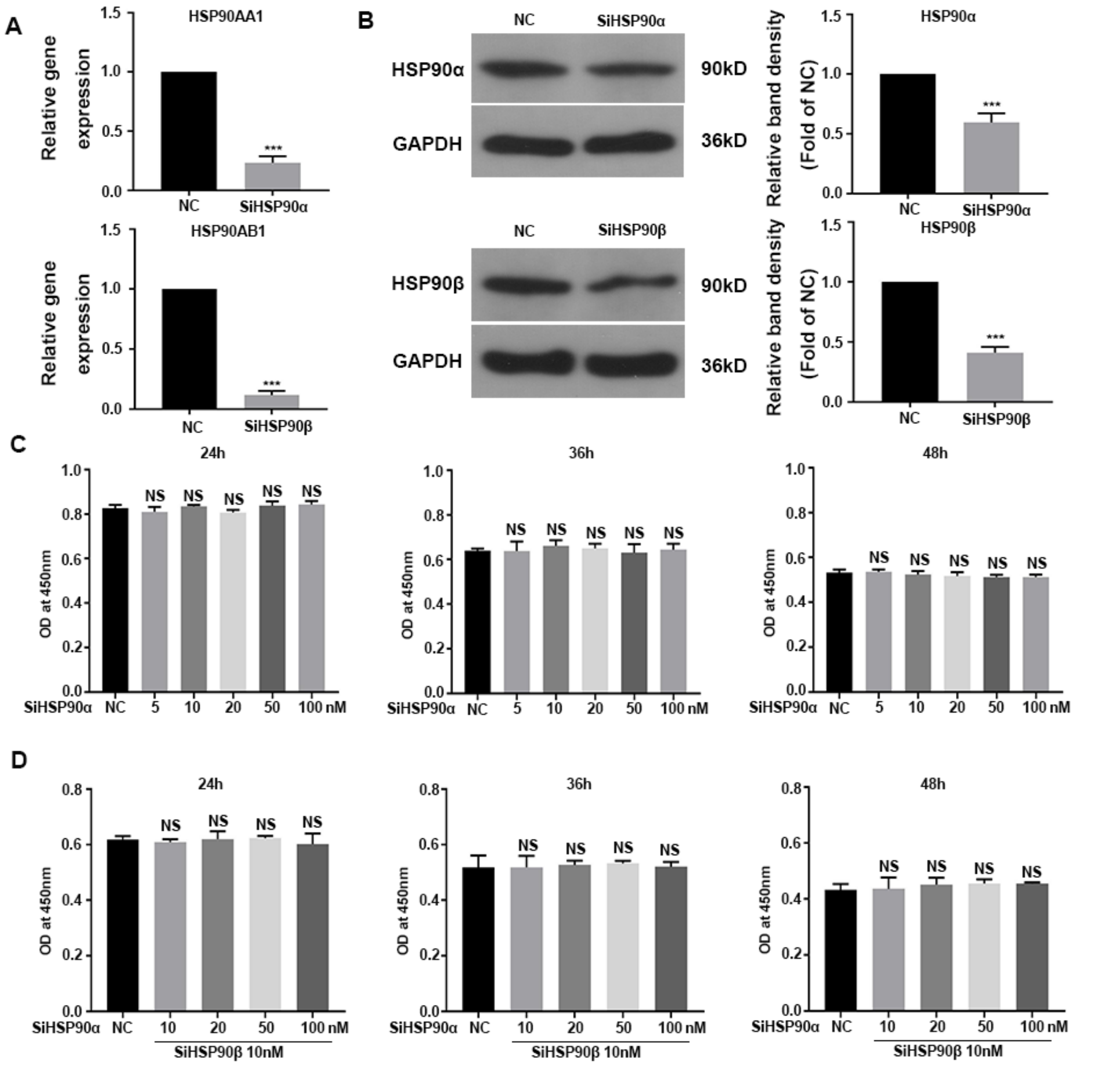

Supplementary Figure 4. Transfection of NPSCs with HSP90α siRNA or both the HSP90α and HSP90β siRNAs has no significant influence on cell viability. **(A)** The transfection efficacy of HSP90α (100nM) and HSP90β (10 nM) siRNAs was validated by RT-PCR. **(B)** Representative WB graphs and quantitation of the expression levels of HSP90α and HSP90β. **(C)** The effects of SiHSP90α on cell viability of NPSCs exposed to 24 h, 36 h and 48 h compression measured by CCK-8 assays. **(D)** The effects of co-transfection of SiHSP90β (10 nM) and different concentrations of SiHSP90α on cell viability of NPSCs exposed to 24 h, 36 h and 48 h compression measured by CCK-8 assays. The data were expressed as mean ± SD from at least three independent experiments, and they were analyzed by a two-tailed t-test. (****P*< 0.001 vs. NC, NS, not significant)

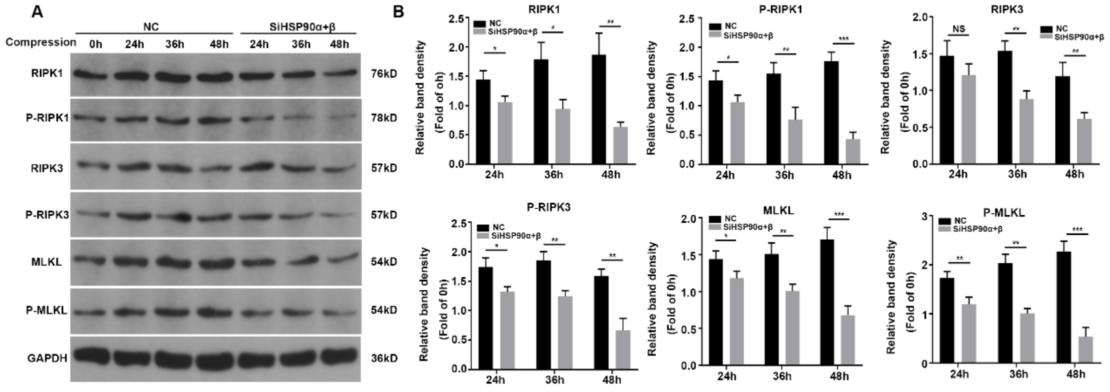

Supplementary Figure 5. The effects of HSP90 specific siRNAs on the expression of core necroptosis regulators. **(A-B)** Representative WB graphs and quantitation of the expression levels of RIPK1, P-RIPK1, RIPK3, P-RIPK3, MLKL and P-MLKL. The data were expressed as mean ± SD from three independent experiments, and they were analyzed by a two-tailed t-test. (**P*< 0.05, ***P*< 0.01, ****P*< 0.001 vs. NC, NS, not significant).
